# Urologists are optimistic surgeons: prevalence and predictors of discordance between intraoperative stone-free rate and cross-sectional imaging evaluation after vacuum-assisted mini-percutaneous nephrolithotomy

**DOI:** 10.1007/s00345-022-04091-3

**Published:** 2022-07-13

**Authors:** Letizia Maria Ippolita Jannello, Matteo Turetti, Carlo Silvani, Gilda Galbiati, Susanna Garbagnati, Efrem Pozzi, Matteo Malfatto, Stefano Paolo Zanetti, Fabrizio Longo, Elisa De Lorenzis, Giancarlo Albo, Andrea Salonia, Emanuele Montanari, Luca Boeri

**Affiliations:** 1 Department of Urology, Foundation IRCCS Ca’ Granda, Ospedale Maggiore Policlinico, Via della Commenda 15, 20122 Milan, Italy; 2 Department of Clinical Sciences and Community Health, University of Milan, Milan, Italy; 3 Division of Experimental Oncology/Unit of Urology, URIIRCCS Ospedale San RaffaeleUniversity Vita-Salute San Raffaele, Milan, Italy

**Keywords:** Percutaneous nephrolithotomy, Vacuum-assisted percutaneous nephrolithotomy, Stone-free rate, Intraoperative evaluation

## Abstract

**Purpose:**

To assess how accurate are urologists in predicting stone-free status (SFS) after vacuum-assisted mini-PCNL (vamPCNL) compared to computed tomography (CT) and clinical predictors of discordant SFS.

**Methods:**

Data from 235 patients who underwent vamPCNL were analysed. Patient’s demographics, stones’ characteristics and operative data were recorded. SFS was evaluated intraoperatively by the treating urologist (iSFS) and with non-contrast CT 3 months after vamPCNL (ctSFS). SFS was defined as no residual stones. Stone complexity was scored with the Guy’s score. Descriptive statistics and logistic regression models were used to identify clinical factors associated with discordant SFS (namely iSFS not confirmed at CT).

**Results:**

iSFS and ctSFS were 88.5% and 65.5%, respectively, with 54 (23%) cases resulting in discordant evaluation of SFS between the surgeon and CT imaging. Patients with discordant SFS had larger stone volume (*p* < 0.001), higher rate of multiple stones (*p* = 0.03) and higher rate of multiple calyceal groups affected by stones (*p* < 0.001) than those with concordant SFS. The use of flexible ureteroscopes to look for residual stones after lithotripsy was more frequently reported in cases with concordant SFS (*p* = 0.001). Multivariable logistic regression analysis revealed that stones in > 2 calyceal groups (OR 10.2, *p* < 0.001), Guy’s score II (OR 5.8, *p* < 0.01) and not using flexible ureteroscopes after lithotripsy (OR 2.9, *p* = 0.02) were independent predictors of discordant SFS.

**Conclusion:**

One out of five patients is erroneously considered SF after vamPCNL. Urologist should carefully evaluate patients with multiple calyceal stones and consider using flexible ureteroscopes to complete lapaxy of migrated fragments in order to improve their prediction of SFS.

## Introduction

Urolithiasis incidence is increasing along with world wellness, with a prevalence rising in the last decades to roughly 10% of the population in developed countries and 20–25% of the population in the Middle East [1]. Many are the factors involved in the development of stones, such as metabolic disorder, genetic predisposition, intake of drugs stone-inducer, anatomical abnormalities, and environmental and professional factors [2, 3]. For better stratification of patients and more personalized management, each of these factors should be analyzed. Management options are active surveillance, medical expulsive therapy, chemolysis or surgical intervention with a rate that vary from 7 to 27% [3, 4].

According to the most recent European Association of Urology (EAU) Guidelines on urolithiasis, percutaneous nephrolithotomy (PCNL) is the gold-standard procedure for large stones in adult population [3]. Despite PCNL has shown to be highly effective in stone clearance [5], it might be associated with severe complications, thus including fever, bleeding and sepsis [6, 7].

In the past decades, the miniaturization of PCNL technique have managed to optimize the need for a minimally invasive procedure while maintaining an equal efficiency, measured as stone-free rate, and comparable, if not lower, postoperative complications as compared to standard procedures [8–10]. Mini-PCNL (mPCNL) has still some limitations as smaller tract sizes may have a negative impact on the operative time, decreased visibility with difficulty in stone retrieval and increased intraoperative renal pressures, with consequent higher rates of infectious complications [11]. To overcome those drawbacks, innovative methods have achieved the aim of a continuous irrigations flow in low-pressure conditions, maximizing the benefit of a minimally invasive procedure using the so-called “vacuum-assisted” system [12, 13].

One of the main outcomes of PCNL is stone-free status (SFS). Over the years, there has been a major divergence in the definition of SFS; nonetheless, each study has stressed the importance of unification [14]. In clinical practice, no residual stones or residual fragments ≤ 4 mm detected at computer tomography (CT) scan after the procedures are the most widely used definitions of SFS [14–16]. Intraoperative stone-free status, as by surgeon’s judgment, is widely used as a surrogate for treatment efficacy [17]. Moreover, it is used in clinical practice to guide the decision over the postoperative management of patients undergoing PCNL (e.g., oral chemolysis, follow-up imaging). However, few studies have investigated the correlation between intraoperative SFS, as defined by surgeon’s judgment, and the gold-standard evaluation with CT postoperatively [18]. In particular, this topic has never been evaluated in vacuum-assisted PCNL (vamPCNL) which is the most recent evolution of PCNL armamentarium, and it has been associated with higher SFS than classic PCNL [13, 19].

Thereof, this study aimed to assess the rate of concordance between intraoperative SFS (iSFS) and CT-based SFS (ctSFS) after vamPCNL and clinical predictors of discordance between urologist’s evaluation and CT imaging in a cohort of patients with kidney stones.

## Materials and methods

Data prospectively collected from 297 patients who underwent vamPCNL in our tertiary-referral academic center between June 2016 and December 2021 were retrospectively analysed.

We recorded patients’ demographics and comorbidities, scored with the Charlson comorbidity index (CCI) [20]. The CCI was further categorized as 0 vs. ≥ 1. A urographic CT scan was performed before surgery in each case. Stone characteristics such as stone volume, location, side, burden (single or multiple), stone density (Hounsfield unit—HU) [21, 22] and the number of calyceal groups affected by stones were recorded. Stone volume was calculated using the ellipsoid formula (length × width × height × π × 1/6) [23].

### Surgical procedures

All the procedures were performed under general anesthesia with the patient in the supine Valdivia position by two expert endourologists (E.M; F.L). The 16 Ch ClearPetra set (namely, vamPCNL), the 12 Ch minimally invasive nephroscope [24] (Karls Storz, Tuttlingen, Germany) and the holmium laser (VersaPulse PowerSuite 100 W, Lumenis, Israel) were used. First, a ureteral catheter was placed in the renal pelvis to inject contrast medium. Renal puncture was performed under fluoroscopic and ultrasonographic control. Stones were treated with a 550 μm holmium:YAG laser fiber for fragmentation, with settings according to surgical needs, and then evacuated through the aspiration-assisted sheath in fragments [13]. The aspiration pressure could be regulated throughout the procedure. Flexible ureteroscope (7.9 Fr, Olympus URF-P6, Germany) and nitinol baskets were used through the percutaneous access when residual fragments could not be removed with the vacuum-assisted device. A final fluoroscopy with contrast medium injection and washout was performed in every patient after lithotripsy to look for collecting system leakage or residual fragments. As exit strategy, an 8 Ch nephrostomy tube was placed in all cases, while the ureteral catheter was either left in place or removed at the end of the procedure based on the surgeon’s preference. At the end of the procedure, intraoperative SFS (iSFS) was judged by the operating surgeon. These data were reported both in the operating record and in a dedicated questionnaire that contains other intraoperative variables (e.g., type of laser fiber, laser energy, number of access tracts) that are prospectively collected and included in a dedicated database.

### Postoperative setting

According to our internal protocol, uncomplicated procedures were managed as follows: the bladder catheter was removed on postoperative day one and the nephrostomy tube was closed; on postoperative day two a percutaneous pyelography was performed to assess ureteral canalization and the presence of residual stones. If ureteral canalization was confirmed, the nephrostomy tube was removed. Patients were discharged on postoperative day three.

The Guy’s stone score was used to grade the complexity of vamPCNL [25]. Postoperative complications were graded according to the PCNL-adjusted Clavien Score [26, 27]. Patients were evaluated within 3 months after surgery with non-contrast enhanced CT scan to identify residual stones. The CT-based postoperative stone-free rate (ctSFR) was defined as the absence of residual fragments [17]. Patients with residual fragments were offered, according to stone dimension, observation or auxiliary procedures including second-look PCNL, extracorporeal shockwave lithotripsy, or retrograde intrarenal surgery.

We excluded patients with renal or skeletal anomalies (*N* = 21); scheduled staged procedures for large stone burden (*N* = 42); endoscopic combined intrarenal surgery procedures (*N* = 3). A final cohort of 235 patients who underwent vamPCNL for kidney stones was considered for statistical analysis.

Data collection adheres to the principles of the Declaration of Helsinki. All patients signed an informed consent agreeing to share their own anonymous information for future studies. The study was approved by the Foundation IRCCS Ca’ Granda, Ospedale Maggiore Policlinico Ethical Committee (Prot. 25508).

### Statistical analysis

Distribution of data was tested with the Shapiro–Wilk test. Data are presented as medians (interquartile range; IQR) or frequencies (proportions). Descriptive statistics were used to describe the whole cohort. First, the rate of discordant SFS was recorded (namely, iSFS not confirmed at CT). Second, clinical parameters, intraoperative and postoperative characteristics were compared between participants with concordant and discordant SFS with the Mann–Whitney test and Fisher Exact Test, as indicated. Lastly, univariable and multivariable logistic regression models tested the association between clinical variables and discordant SFS. Statistical analyses were performed using SPSS v.26 (IBM Corp., Armonk, NY, USA). All tests were two sided, and statistical significance level was determined at *p* < 0.05.

## Results

Table 1 details demographic characteristics, intraoperative and postoperative attributes among the whole cohort. Overall, median (IQR) age and BMI were 56 (48–67) years and 24.9 (21.9–27.7) kg/m^2^, respectively. Of 235, 145 (61.5%) patients had multiple stones, with a median stone volume of 1.9 (0.9–3.3) cm^3^. The Guy’s stone score was I, II and III in 90 (38.2%), 95 (40.4%) and 50 (21.4%) patients, respectively. Operative time and length of stay were 95 (75–130) min. and 4 (3–5) days, respectively. iSFS and ctSFS were 88.5% and 65.5%, respectively, with 54 (23%) cases resulting in discordant evaluation of SFS between surgeon and CT imaging.

**Table 1:** Demographic characteristics of the whole cohort (*n* = 235)

Age (years)	
Median (IQR)	56.0 (48–67)
Range	19–84
Male gender [no. (%)]	136 (57.9)
BMI (kg/m^2^)	
Median (IQR)	24.9 (21.9–27.7)
Range	17.9–46.1
CCI (score)	
Median (IQR)	0.0 (0.0)
Mean (SD)	0.5 (0.2)
Range	0–5
CCI ≥ 1 [no. (%)]	85 (36.5)
Laterality [no. (%)]	
Right	110 (46.8)
Left	125 (53.2)
Stone volume (cm^3^)	
Median (IQR)	1.9 (0.9–3.3)
Range	0.5–21.2
Single stone [no. (%)]	90 (38.5)
Stone density (Hounsfield unit)	
Median (IQR)	1230 (850–1415)
Range	122–2286
Number of affected calyces [no. (%)]	
Single or pelvis	166 (70.6)
Multiple	69 (29.4)
Guy’s stone score [no. (%)]	
Grade I	90 (38.2)
Grade II	95 (40.4)
Grade III	50 (21.4)
Operative time (min)	
Median (IQR)	95.0 (75–130)
Range	36–245
Flexible nephroscopy [no. (%)]	139 (59.1)
Hospitalization time (days)	
Median (IQR)	4.0 (3.0–5.0)
Range	2.0–12.0
Postoperative complications [no. (%)]	
Clavien–Dindo I	16 (6.8)
Clavien–Dindo II	33 (14.0)
Clavien–Dindo IIIa/b	12 (5.1)
Intraoperative stone-free rate [no. (%)]	208 (88.5)
CT-based stone-free rate [no. (%)]	154 (65.5)

*BMI* body mass index, *CCI* Charlson Comorbidity Index, *CT* computerized tomography

Table 2 reports perioperative characteristics among participants with concordant vs. discordant stone-free status. Patients with discordant SFS had larger stone volume [2.8 (1.7–5.9) cm^3^ vs. 1.6 (0.8–2.9) cm^3^, *p* < 0.001], higher rate of multiple stones (74.1% vs. 58.1%, *p* = 0.02) and higher rate of multiple calyceal groups affected by stones (44.7% vs. 25.4%, *p* < 0.001) than those with concordant SFS. Similarly, a higher rate of Guy’s score II was found in patients with discordant SFS (56.7% vs. 36.4%).

**Table 2:** Descriptive statistics of the whole cohort as segregated according to discordant stone-free status (*n* = 235)

	Concordant	Discordant	*P* value*
Number of patients [no. (%)]	181 (77.0)	54 (23.0)	
Age (years)			0.4
Median (IQR)	57.0 (49–67)	56.0 (42–65)	
Range	19–84	20–83	
Male gender [no. (%)]	103 (56.9)	33 (61.1)	0.5
BMI (kg/m^2^)			0.5
Median (IQR)	24.9 (22.2–27.9)	24.7 (21.4–27.7)	
Range	17.9–46.1	18.1–42.2	
CCI (score)			0.8
Median (IQR)	0.0 (0.0)	0.0 (0.0)	
Mean (SD)	0.4 (0.2)	0.5 (0.3)	
Range	0–4	0–5	
CCI ≥ 1 [no. (%)]	65 (36.3)	20 (37.0)	0.9
Laterality [no. (%)]			0.3
Right	86 (47.5)	24 (44.4)	
Left	95 (52.5)	30 (55.6)	
Stone volume (cm^3^)			< 0.001
Median (IQR)	1.6 (0.8–2.9)	2.8 (1.7–5.9)	
Range	0.2–21.2	0.5–13.8	
Single stone [no. (%)]	76 (41.9)	14 (25.9)	0.02
Number of affected calyces [no. (%)]			< 0.01
Single or pelvis	135 (74.6)	31 (55.3)	
Multiple	46 (25.4)	25 (44.7)	
Guy’s stone score [No. (%)]			< 0.01
Grade I	79 (43.6)	11 (20.4)	
Grade II	66 (36.4)	29 (53.7)	
Grade III	36 (20.0)	14 (25.9)	
Stone density (Hounsfield unit)			0.6
Median (IQR)	1250 (856–1415)	1200 (763–1425)	
Range	102–2286	100–1810	
Operative time (min)			0.9
Median (IQR)	97.0 (72–130)	92 (78–131)	
Range	36–215	40–210	
Flexible nephroscopy [no. (%)]	118 (65.2)	21 (38.9)	0.01
Hospitalization time (days)			0.06
Median (IQR)	4.0 (3.0–5.0)	5.0 (3.0–7.0)	
Range	2.0–12.0	2.0–12.0	
Postoperative complications [no. (%)]			0.6
Clavien–Dindo I	12 (26.1)	4 (26.7)	
Clavien–Dindo II	24 (52.2)	9 (60.0)	
Clavien–Dindo IIIa/b	10 (21.7)	2 (13.3)	

*BMI* body mass index *CCI* Charlson Comorbidity Index

^*^p value according to the Mann–Whitney test and Fisher Exact test, as indicated

The use of flexible ureteroscopes to look for residual stones after lithotripsy was more frequently reported in cases with concordant SFS (65.2% vs. 38.9%, *p* = 0.001). Groups were similar in terms of age, BMI, CCI and operative time.

Table 3 depicts logistic regression models testing the association between clinical variables and discordant stone-free status. Univariable logistic regression analysis showed that higher stone volume (OR 1.11, 95% CI 1.06–1.68), not using flexible ureteroscopes during surgery (OR 0.34, 95% CI 0.18–0.63), the presence of stones in 2 (OR 3.16, 95% CI 1.04–9.57) and > 2 calyceal groups (OR 9.81, 95% CI 2.49–15.23) and Guy’s stone score II (OR 3.51, 95% CI 1.56–7.89) and III (OR 2.74, 95% CI 1.09–6.87) were all associated with discordant SFS. Similarly, multivariable logistic regression analysis (model 1) revealed that stones in > 2 calyceal groups (OR 10.2, 95% CI 3.16–16.22) and not using flexible ureteroscopes after lithotripsy to look for residual stones (OR 0.34, 95% CI 0.13–0.88) were independent predictors of discordant SFS after accounting for age, stone volume and operative time. Similarly, Guy’s stone score II (OR 5.81, 95% CI 1.92–13.49) was found to be associated with discordant SFS after accounting for the same variables (model 2).

**Table 3:** Logistic regression models predicting discordant stone-free status in the whole cohort

	UVA model OR; *p* value [95% CI]	MVA model OR; *p* value [95% CI]	MVA model OR; *p* value [95% CI]
		Model 1	Model 2
Age	0.99; 0.43 [0.97–1.02]	0.98; 0.54 [0.95–1.03]	0.98; 0.29 [0.94–1.02]
BMI	1.02; 0.84 [0.93–1.47]		
CCI ≥ 1	1.03; 0.92 [0.54–1.94]		
Female gender	0.84; 0.58 [0.45–1.56]		
(vs. male)			
Stone volume	1.11; 0.03 [1.06–1.68]	1.11; 0.1 [0.96–1.25]	1.14; 0.08 [0.99–1.32]
Stone density (HU)	1.01; 0.46 [0.98–1.07]		
Flexible nephroscopy	0.34; 0.01 [0.18–0.63]	0.34; 0.02 [0.13–0.88]	0.38; 0.03 [0.15–0.91]
Operative time	0.99; 0.81 [0.99–1.02]	0.99; 0.3 [0.98–1.06]	0.99; 0.54 [0.98–1.02]
No. of involved calyces			
Single/renal pelvis	Ref	Ref	
2 calyces	3.16; 0.04 [1.04–9.57]	2.91; 0.1 [0.74–11.41]	
> 2 calyces	9.81; < 0.01 [2.49–15.23]	10.2; < 0.001 [3.16–16.22]	
Guy’s stone score			
Grade I	Ref		Ref
Grade II	3.51; 0.02 [1.56–7.89]		5.81; 0.01 [1.92–13.49]
Grade III	2.74; 0.03 [1.09–6.87]		2.98; 0.15 [0.66–11.85]

*UVA* Univariate model, *MVA* Multivariate model, *BMI* body mass index, *CCI* Charlson Comorbidity Index, *HU* Hounsfield unit

## Discussion

This study was specifically designed to investigate the rate of and predictors of discordance between intraoperative SFS, as for surgeon’s estimation, and CT-based SFS after vamPCNL. We found that the surgeon was able to correctly predict SFS in 77% of cases after surgery and that stones in multiple calyceal groups, procedural complexity as well as the lack of flexible nephroscopy to look for residual stones after lithotripsy were independent predictors for discordant SFS.

The study was motivated by the substantial lack of research concerning the surgeon’s ability to predict SFR in vamPCNL, which is the one of the latest technical evolution of mPCNL [10]. In particular, previous studies have shown that vamPCNL was associated with higher SFS than classic mPCNL [12, 13] but the precision of iSFS compared to ctSFS has never been evaluated.

The intraoperative surgeon perspective of SFS after classic PCNL compared to CT-based evaluation was reported for standard PCNL. Harratz et al. analysed data from 306 patients submitted to PCNL and found that 72% of cases were deemed stone free by the operating surgeon and subsequently confirmed after CT scan [28]. Authors also revealed that procedural complexity, as defined by the Guys score, was significantly associated with the true negative surgeon’s opinion of stone free after surgery, thus suggesting that iSFS was reliable primarily in patients with simple stones [28]. Our study confirms these findings since we found that Guy’s score II was associated with discordant SFS. Similarly, Portis et al. performed a prospective study on 39 kidney units undergoing PCNL and showed that 26.5% of cases had residual fragments at postoperative imaging despite being considered stone free during surgery [29]. More recently, a larger study with 312 classic PCNL performed between 2010 and 2015, reported a 80% concordance rate between iSFS and ctSFS [18]. Multiples stones and the cumulative stone size were found to be independent predictors for missed SFS evaluation [18]. Our results are concordat with data in published literature since we found that urologists can accurately predict SFS in 77% of cases after vamPCNL. In our series, one out of five patients is defined SF intraoperatively but showed residual fragments at CT follow-up. Of note, the presence of stones in multiple calyceal groups and omitting flexible nephroscopy to look for residual stones after lithotripsy were found to be independent predictors for discordant SFS.

Our results confirm the intuitive thinking that with increasing number of calyces involved, the likelihood of a missing residual fragments is higher. This was confirmed by previous studies showing that the higher the number of stone the higher the probability of being non-stone free after surgery [18, 30]. Of note, our results suggest that patients with multiple stones (Guy’s score II) were at higher risk of missing residual fragments compared to those with single (Guy’s score I) or partial staghorn stone (Guy’s III). Once again, this study reveals that the number of calyces involved was a better prognosticator for discordant SFR as compared to stone volume in vamPCNL. Of clinical importance, we showed that flexible nephroscopy after lithotripsy was important for achieving SFS. However, flexible nephroscope is not routinely performed in every institution, particularly after mPCNL [31]. Complex renal stones that require prolonged disintegration might result in many fragments that are dispersed in peripheral calyces during the procedure. Gucuk et al. [32] compared data from a group of patients treated with standard PCNL and a group treated with PCNL and flexible nephroscopy at the end of the procedure. They found that SFS was higher in PCNL performed with flexible nephroscopy. In another study, anterograde vs. retrograde endoscopy to complete the PCNL were compared [33]. They demonstrated the benefit of matching the two modalities in terms of SFS, with particular advantages of the retrograde approach. However, they did not discriminate which patients would benefit more from one approach compared to the other. One of the major advantages of vamPCNL is the continuous aspiration of fragments during lithotripsy that reduces operative time and limits stone migration [13]. In this context, surgeons could potentially avoid the use of flexible nephroscopy and additional instruments (baskets or grasper) in vamPCNL in order to reduce hospitalization costs [19]. Our results suggest that flexible nephroscopy is useful even during vamPCNL, with specific role in patients with multiple calyces involved by stones.

The importance of our study is several folds. First, we showed that urologist can intraoperatively predict SFS in 77% of cases during vamPCNL, with important clinical implications in terms of patient’s postoperative management. For instance, since a significant proportion of patients with residual fragments after PCNL requires further interventions, the surgeon’s assessment of residual stones immediate after the procedure is of critical importance when a tubeless approach is contemplated [17, 34]. Furthermore, in clinical practice, routine postoperative imaging is decided according to stone and procedural characteristics. It is common that surgeon’s opinion is used as a tool to mitigate postoperative imaging, with consequent reduction in radiation exposure to patients and costs [35, 36]. Second, we showed that iSFS was more accurate in patients with single or partial staghorn stones vs. multiple calculus and if flexible nephroscopy was performed to look for residual fragments. Therefore, it could be speculated that in vamPCNL procedures, characterized by shorter operative time and higher SFS than classic mPCNL [13], the addition of flexible nephroscopy would make this surgery even more precise and accurate.

This study is not devoid of limitations. Our definition of SFS as “no stones” is not universally adopted [17]. However, nowadays, there is yet not a unified definition and we aimed to be more rigorous in terms of PCNL outcomes. In fact, it should be mentioned that SFR in our series was lower compared to that reported by different authors for the treatment of kidney stones with mPCNL (range 80–95%) [37, 38]. However, in the previous series, the authors considered SF also cases with residual fragments of < 4 mm or used plain X-ray for evaluation, thus partially explaining the difference in SFR with our series. This is a single center-based and retrospective study, which raises the possibility of selection biases. Thereof, larger prospective studies across different centers and cohorts are needed to externally validate our findings. Finally, all PCNL were performed in a tertiary-referral center for complex stone surgery, therefore, our results cannot be generalized to minor centers.

## Conclusions

In clinical practice, one out of five patients is erroneously considered stone free after vamPCNL. Stones in multiple calyceal groups and the lack of flexible nephroscopy to look for residual stones after lithotripsy were independent predictors for discordance between intraoperative and CT-based SFS. Urologist should carefully evaluate patients with multiple calyceal stones and consider routine use of flexible nephroscopy to retrieve migrated fragments in order to improve their prediction of SFS.
